# BS-CP: Efficient streaming Bayesian tensor decomposition method via assumed density filtering

**DOI:** 10.1371/journal.pone.0312723

**Published:** 2024-12-02

**Authors:** Jiaqi Liu, Qiwu Wu, Lingzhi Jiang, Renjun Zhan, Xiaochuan Zhao, Husheng Wu, Weicong Tan

**Affiliations:** 1 School of Information Engineering, Engineering University of People’s Armed Police of China, Xi’an, China;; 2 Engineering University of People’s Armed Police of China, Xi’an, China; 3 Institute of Computer Application Technology, China North Industries Group, Beijing, China; Northwestern Polytechnical University, CHINA

## Abstract

Tensor data is common in real-world applications, such as recommendation system and air quality monitoring. But such data is often sparse, noisy, and fast produced. CANDECOMP/PARAFAC (CP) is a popular tensor decomposition model, which is both theoretically advantageous and numerically stable. However, learning the CP model in a Bayesian framework, though promising to handle data sparsity and noise, is computationally challenging, especially with fast produced data streams. The fundamental problem addressed by the paper is mainly tackles the efficient processing of streaming tensor data. In this work, we propose BS-CP, a quick and accurate structure to dynamically update the posterior of latent factors when a new observation tensor is received. We first present the BS-CP1 algorithm, which is an efficient implementation using assumed density filtering (ADF). In addition, we propose BS-CP2 algorithm, using Gauss–Laguerre quadrature method to integrate the noise effect which shows better empirical result. We tested BS-CP1 and BS-CP2 on generic real recommendation system datasets, including Beijing-15k, Beijing-20k, MovieLens-1m and Fit Record. Compared with state-of-the-art methods, BS-CP1 achieve 31.8% and 33.3% RMSE improvement in the last two datasets, with a similar trend observed for BS-CP2. This evidence proves that our algorithm has better results on large datasets and is more suitable for real-world scenarios. Compared with most other comparison methods, our approach has demonstrated an improvement of over 10% and exhibits superior stability.

## Introduction

Tensors are multidimensional arrays, which naturally represent multiple modes or dimensional information. Tensor decomposition gives us the structure to analyse this information, by minimizing the reconstruction error of the observed tensor values to estimate a set of latent factors. From these factors, we can discover hidden patterns in data. In the meantime, these factors can be used as informative features in downstream tasks. Therefore, tensor decomposition is popular in many practical applications, such as weather prediction, medicine treatment effect prediction, and recommendation system. For example, we can build a three dimensions tensor data from people’s fitness records of (user, sport type, altitude), or we can get four mode tensor data from patients’ vaccination status like (area, patient, brand, time). More traditionally, we could take user ratings from an online platform and build tensor data, various from movies (MovieLens) to games (Steam video games).

CANDECOMP/PARAFAC (CP) is a widely used tensor decomposition method [1]. It splits a high-dimensional tensor into the sum of multiple rank-1 tensor, each of which is formed by a vector outer product. All tensor nodes have the same number of potential factors *R*. In each *r* of *R*, the *r*-th product is derived by computing the *r*-th latent factor of each involved node. In this way, we can significantly reduce the dimension of the parameter. Other commonly used decomposition methods such as tucker decomposition [2] and block term decomposition (BTD) [3], the former is a complicated version of CP, it can be seen as a higher-order version of principal component analysis (PCA), which decomposes a tensor into core tensors multiplied by the corresponding matrix on each dimension. The difference between CP and tucker is that the size of their core tensor, tucker tensor core has the size of *R*_1_ × *R*_2_ × *R*_3_ × … × *R*_*n*_, while CP tensor core has the size of *R* × *R* × *R* × … × *R*. BTD decomposition can be seen as a higher generalization of CP decomposition and tucker decomposition, assumes that a decomposition of *Rank* − (*L*_*r*_, *M*_*r*_, *N*_*r*_), each member tensor is a *Rank* − (*L*_*r*_, *M*_*r*_, *N*_*r*_) Tucker decomposition. It decomposes a *Rank* − (*L*_*r*_, *M*_*r*_, *N*_*r*_) tensor into *R* member tensors. When *R*=1, there is only one member tensors, and this decomposition is a Tucker decomposition. When each member tensor is a Rank-1 decomposition, it reduced to a CP decomposition.

It can be seen that CP decomposition in S1(a) Fig enables the original tensor to be represented by multiple vectors, while Tucker decomposition in S1(b) Fig also retains a core tensor with the same dimension as the original tensor, and the dimension of the core tensor increases with the increase of the dimension of the original tensor, making the decomposed factors more complex. Despite the generalization ability due to their high complexity, given the relatively large core tensor in tucker decomposition and BTD decomposition, model estimates are much more expensive and cumbersome than CP, and because of the similar structure, CP decomposition would be a more economical and fast solution. In addition, when a batch of new entries is received, it is very costly to estimate the core tensor, let alone a rather large and indeterminate core tensor.

Classical tensor decomposition methods involve using alternate least squares (ALS) [4] or gradient optimization methods [5] to update the embeddings. However, these methods can only work on the static data, also, they need the whole dataset to perform each update operation. Processing tensor data in a streaming fashion has become a trend recently due to its highly time-efficiency [6, 7]. In this scenario, the target is to calculate the posterior distribution of the embeddings along with the incoming of the data. There are several ways of computing posterior distributions, such as Markov Chain Monte Carlo (MCMC) [8], Laplace’s method [9], variational Bayes (or variational inference) [10], etc. Assumed density filtering (ADF) is a single-order method for calculating an approximate posterior distribution [11]. Its key advantage is that it does not require linearization of the system model therefore suitable for highly nonlinear systems.

However, because of the calculation of integrals in high-dimensional spaces, ADF may be difficult to implement on higher dimensional space system or limited computational resources. In addition, due to its sequential feature, information discarded early may become important later. ADF is also sensitive to observation sorting, which is undesirable in a batch processing context. And, with limited computing resources, it can be difficult for ADF to achieve fast convergence and high accuracy.

In summary, there are several existing problems with approaches using tensor factorization in recommendation algorithms:

1. Facing the real-time streaming data on the Internet today, the method based on static data acquisition usually takes a long time to retrain or update the model, which cannot meet our needs in time.For example, the product recommendation system of an e-commerce platform needs to provide personalized recommendations based on users’ real-time browsing and purchase behavior. If the recommendation system is built on the basis of a user’s past purchase history, it may not well reflect the user’s latest interests and needs, which can affect the user experience and the platform’s sales performance. Similarly, companies need to monitor changes in sentiment on social media in order to respond to public opinion in a timely manner when conducting brand management. Sentiment analysis models based on static data sets may not be able to capture the mood swings caused by unexpected events or hot topics, resulting in enterprises unable to adjust market strategies in a timely manner.
2. Using methods such as deep learning makes the model lack interpretability and thus cannot explain its recommendation reasons well to the user. For example, in the medical field, if the model does not explain why an image is identified as a tumor, doctors may be skeptical of the model’s predictions, and this opacity may lead doctors to distrust AI-assisted systems, thereby affecting patient care. Moreover, a user may be recommended a completely unrelated item after browsing some products about fitness, and if the user does not understand the logic of the recommendation, it may cast doubt on the effectiveness of the recommendation system.
3. The accuracy of the algorithm needs to be improved. Tensor factorization models may be sensitive to noise in the data, which may lead to inaccurate recommendation results. Also, the accuracy of the mathematical calculation method itself improved to match more diverse user needs. For example, if the data set contains false or untrue ratings (noisy data), such as an extreme rating given by a user because of personal mood swings, the tensor decomposition model may incorrectly include these extreme ratings in the analysis, causing the model to capture the wrong user preferences and thus recommend irrelevant movies. In terms of improving the accuracy of the algorithm, the e-commerce platform provides users with personalized product recommendations through the recommendation system. If the algorithm of the recommendation system is low in precision and cannot accurately predict the user’s preferences, it may lead to poor user experience and users cannot quickly find the goods they need, thus affecting sales and customer satisfaction.

To address these problems, we develop BS-CP, a Bayesian Streaming CP decomposition approach. We use moment matching method according to [12] to address the intractable moment calculation in ADF framework, and develop a one-shot incremental posterior update for the latent factors upon receiving each streaming batch. Because our approach, unlike the black box scenario of deep learning, uses a machine learning approach based on Bayesian probability inference, the algorithm is interpretable, such as the e-commerce recommendation system, which can explain the reason for the recommendation well to the user. It provides a more computationally efficient method, approximating the result of state estimation by matching only the first and second order moments. The structural diagram of the BS-CP algorithm is presented in S2 Fig. It can improve the state estimation of the ADF by matching higher order moments. We name a simplified implementation as BS-CP1. Furthermore, noticing that the exponential function is needed in each calculation of posterior update, we use Gauss-Laguerre quadrature [13] to simplify operations. Gauss-Laguerre quadrature uses specific weights and nodes, which can obtain higher integration accuracy with less computation, making the integration process simpler and more stable. These weights and nodes are pre-calculated to minimize integration errors. For exponential functions, Gauss-Laguerre quadrature converges faster, so it is usually faster than other numerical integration methods when calculating the integral of such functions. We summarize it in BS-CP2.

To evaluate our method, we used four real-world datasets to examine BS-CP, including both small and large-size tensors. For comparison, we chose state-of-the-art streaming decomposition algorithm POST [6], BASS-Trucker [14] and the time-information-added streaming algorithm CT-CP [15]. In terms of operation and final predicted performance, our approach consistently outperforms competing methods. In addition, from the perspective of accuracy, BS-CP algorithm is more stable than the competing methods.

## Related work

### Tensor decomposition

Many methods have been devoted to compute tensor decomposition, as a classic factorization technique, CP decomposition [1] has been studied from many angles, such as CP-ALS [16–18], MAST [19], POST [6], tucker decomposition is also used, such as Tucker-ALS [4], pTucker [20], InfTucker [21, 22], BPTD [23], TGP [24], BASS-Tucker [14]. There are other decomposition methods, such as singular value decomposition (SVD) [25], tensor train decomposition (TTD) [26] and tensor rain decomposition (TRD) [27], etc. Although there are many methods have been used for tensor decomposition, the CP and tucker decomposition methods are the most widely used, and between the two of them, CP decomposition is used more frequently due to its elegance structure and good interpretability. There are some works aimed at solving the reconstruction problem of the CP decomposition model, for example, Bai et al. [28] developed a new relaxed augmented Lagrangian method for solving a series of convex optimization problems subject to equality or inequality constraints. the augmented Lagrangian method or the involved splitting method in this method can also solve the reformulation problem of CP Tensor Decomposition Model. The aforementioned previous works include both static [4, 17–21, 23], etc. and streaming [6, 7, 26], etc. methods. The streaming data processing method is more practical in the current recommendation system algorithm because of its advantages of timeliness. For example, apps like Instagram, their data will not be stored after it has been accessed. which means we don’t have access to any data other than what we have at this moment and need to update the embed and the model immediately based on the current receiving data. Therefore is necessary to construct a good streaming data processing method, which can be applied to a wider range of recommendation systems. We list the published information on tensor decomposition algorithms in Table 1.

**Table 1:** Information on published tensor decomposition algorithms.

	publication year	publication journal(conference)	decomposition	data form
**Tucker-ALS**	2008	SIAM	Tucker	static
**CP-ALS**	2009	SLAM	CP	static
**pTucker**	2009	AISTATS 2009	Tucker	static
**CP-WOPT**	2011	Elsevier	CP	static
**InfTucker**	2012	ICML 2012	Tucker	static
**BPTD**	2016	ICML 2016	Tucker	static
**TGP**	2016	ArXiv	Tucker	static
**MAST**	2017	KDD 2017	CP	static
**POST**	2018	ICDM 2018	CP	stream
**CEP**	2019	UAI 2019	CP	stream
**STRD**	2019	AAAI 2019	Tensor Rain	stream
**BASS-Tucker**	2021	UAI 2021	Tucker	stream
**CT-CP**	2021	Microtome	CP	stream
**BCTT**	2022	ICML 2022	CP	stream
**STTD**	2023	ArXiv	Tensor Train	stream

### Bayes streaming algorithm

Streaming algorithm [29] is a kind of data processing method, which emphasizes the real-time and continuity of data. In streaming computing, data is continuously input into the system in the form of a stream and processed immediately. This method is suitable for processing large-scale, high-speed data flow, real-time analysis, real-time feedback and other scenarios. Bayesian streaming data processing [30] is a statistical learning technique for processing real-time data streams, which combines the principles of Bayesian inference and online learning (i.e., real-time learning). Streaming data processing involves a series of related work, such as the application of Bayesian networks in real-time systems, the development of online learning algorithms, and the application of time series analysis in streaming data processing. There have been several recent papers that attempt to add continues-time factor into streaming Bayesian data processing, Zhang et al., model a time function as the CP coefficients by using polynomial splines, namely the dynamic tensor recommendation system (CT-CP) [15]. BCTT [7], proposed by Fang et al., conducted continuous-time Tucker decomposition, and built the tensor-core as a function of time. Adding continuous-time to the model can capture complex time relationships between tensor nodes, but their accuracy are not good enough. We will later compare the representative algorithms CT-CP with our model. The concept of “Bayesian streaming data” and the corresponding processing technology have been discussed in many articles, such as Mardani et al. [31], Zhou et al. [32], POST, BASS. In order to solve the problem of minimizing the KL-divergence between real and estimated posterior, according to Thomas P Minka [33], there are several solutions: Laplace’s method [9], Markov Chain Monte Carlo (MCMC) [8], variational Bayes (or variational inference) [10], (Streaming variational Bayes (SVB) in streaming structure [34]), Assumed density filtering (ADF) [11]. In ADF, observations are processed one by one and then the posterior distribution is updated to approximate the posterior distribution before processing the next observation. Minka posted expectation propagation (EP) [33], which is an extension to ADF, removed the sequential nature of it. To process moment calculation, moment matching [35] method is proposed. However, sometimes it can be difficult to compute moments, to solve this problem, Wang and Zhe [36] proposed conditional EP (CEP), using conditional moment matching and Taylor approximation method. Similar to that, our algorithm is also an improvement of moment matching, using Gauss-Laguerre quadrature [13] to compute the hard-to-compute normalized function Z that appears in the moment match, Gauss-Laguerre quadrature method uses its own orthogonality to simplify the calculation of exponential integral and improve the convergence speed and accuracy of the whole model.

### BS-CP compared to other methods

Most of the above methods use CP decomposition and Tucker decomposition techniques, as well as decomposition methods using SVD, tensor train and tensor ring decomposition(STTD [26] and STRD [27]). Notably, CP decomposition and Tucker decomposition are popular due to their intuitive mathematical formulas and stable numerical calculations. Specifically, CP decomposition represents a tensor as the sum of multiple rank-1 tensors, providing a more streamlined model and improving processing efficiency; Therefore, our BS-CP method utilizes CP decomposition. On the contrary, Tucker-ALS, pTucker, InfTucker, BPTD, TGP, BASS-Tucker algorithm uses Tucker decomposition framework and requires more complex post-decomposition processing to adapt to static target analysis and streaming data applications. In an online e-commerce recommendation system, real-time dynamic data must be processed, rather than just focusing on static data(Tucker-ALS, CP-ALS, pTucker, CP-WOPT, InfTucker, BPGD, TGP, MAST) sets that require a large amount of storage capacity. Therefore, BS-CP adopt a streaming data processing approach to effectively address contemporary needs.

Compared with traditional batch learning methods, our BS-CP method, using Bayesian online data processing technology helps to adapt to new information in real time. When juxtaposed with other online learning methods, these methods provide probabilistic interpretations of model parameters while quantifying the uncertainty of predictions. Frequency-based statistical methods often lack the ability to integrate both prior knowledge and empirical data at the same time, and the advantage of Bayesian methods in this regard is that they naturally blend the two elements. In addition, compared to deep learning frameworks characterized by opaque internal mechanisms, Bayesian online strategies provide clear probabilistic insights into the behavior of parameters.

The ADF method we employ allows greater flexibility across different posterior distribution shapes than the Laplacian technique; In addition, ADF avoids the iterative sampling required by MCMC for a posterior approximation. This makes ADF particularly suitable for non-parametric models, compared to VB or VI, which tend to favor parametric structures; In addition, compared to SVB methods, ADF facilitates more seamless online learning through continuous model updates as new data becomes available, a process that is complicated in SVB frameworks, such as the POST algorithm. Extensions such as EP and CEP [36] build on ADF methods; Although CEP employs moment matching and Taylor approximation techniques similar to ours, the mathematical complexity of the latter presents a challenge in implementation, while BS-CP utilize Gauss-Laguerre orthogonality to simplify the index calculation.

Recently developed methods for integrating temporal information into tensor decomposition (e.g., CT-CP [15] and BCTT [7]) have demonstrated improved capabilities in capturing trends and patterns inherent in time-varying datasets—illustrated by applications such as stock market analysis or financial forecasting, where temporal dynamics play a crucial role. However, in scenarios where time is not the most important or where there is no time in the dataset feature, the relevance of time considerations can be significantly reduced depending on the particular application context and underlying data attributes.

## CP tensor decomposition model

Consider a *K*-mode tensor Y in $R^{d_{1}\times \ldots \times d_{K}}$. We aim to estimate *K* factor tensors, $U^{{}^{\circ}}=\left\{U^{1},\ldots ,U^{K}\right\}$, an *R* dimensional vector $u_{j}^{k}$ represent each object *j* in mode *k*. A *d*_*k*_ × *R* embedding matrix can then be built by stacking all the embedding vectors in mode *k*, we have $U^{k}={\left[u_{1}^{k},\ldots ,u_{d_{k}}^{k}\right]}^{⊤}$. Suppose we have collected a dataset of real-valued tensor entry values, $D=\{\left(i_{1},y_{1}\right),\ldots \left(i_{N},y_{N}\right)\}$ where each **i**_*n*_ = (*i*_*n*1_, …, *i*_*nK*_). We can easily obtain from the vectorized form that for each observed entry **i**_*n*_,

$$\begin{array}{c} y_{n}\approx 1^{⊤}\left(u_{i_{n1}}^{1}{}^{\circ}\cdots {}^{\circ}u_{i_{nK}}^{K}\right) \end{array}$$ (1)

where **1** is the vector full of ones, ° performs element-wise multiplication.

Based on CANDECOMP/PARAFAC (CP) decomposition, we use Bayesian model to sample the continuous observed tensor entries. The Bayesian network diagram of CP decomposition is shown in S4 Fig. Placing a standard Gaussian prior over $U^{{}^{\circ}}$,

$$\begin{array}{c} p\left(U^{{}^{\circ}}\right)=\prod_{k=1}^{K}\prod_{j=1}^{d_{k}}\prod_{r=1}^{R}N\left(u_{jr}^{k}|\mu_{jr}^{k},v_{jr}^{k}\right) \end{array}$$ (2)

Where $u_{jr}^{k}\overset{\Delta }{=}u_{j}^{k}\left(r\right)$. Each embedding vector $u_{j}^{k}$ is first sampled from a Gaussian prior distribution. We model the likelihood for each data point *n* with

$$\begin{array}{c} p\left(y_{n}|U^{{}^{\circ}},\tau \right)=N(y_{n}|1^{⊤}\left(u_{i_{n1}}^{1}{}^{\circ}\cdots {}^{\circ}u_{i_{nK}}^{K}\right),\tau^{-1}) \end{array}$$ (3)

where *τ* is the inverse variance. We further place a Gamma prior over *τ*,

$$\begin{array}{c} p\left(\tau \right)=\text{Gam}\left(\tau |a_{0},b_{0}\right), \end{array}$$ (4)

where we can set *a*_0_ = *b*_0_ = 10^−3^ to obtain a diffuse prior. Our joint probability distribution is given by

$$\begin{array}{c} p\left(U^{{}^{\circ}},\tau ,D\right)=p\left(U^{{}^{\circ}}\right)p\left(\tau \right)\prod_{n=1}^{N}p\left(y_{n}|U^{{}^{\circ}},\tau \right). \end{array}$$ (5)

## Online bayesian streaming algorithm

### Streaming structure

Now, we consider the data is produced in a streaming fashion as shown in S3 Fig. Denote by $D_{n-1}$ the data accumulated up to step *n* − 1, and by $p(U^{{}^{\circ}},\tau |D_{n-1})$ the posterior estimated by our model. Currently, let us assume at step *n*, we receive a single entry (**i**_*n*_, *y*_*n*_) (we will extend it to a batch of entries later). Our goal is then to update the posterior, which according to the Bayes’ rule, is

$$\begin{array}{c} p\left(U^{{}^{\circ}}|D_{n}\right)\propto p\left(U^{{}^{\circ}},\tau |D_{n-1}\right)p\left(y_{n}|U^{{}^{\circ}},\tau \right). \end{array}$$ (6)

To enable tractable posterior update, we introduce a fully factorized approximation to the current posterior, which is consistent with the prior,

$$\begin{array}{c} p\left(U^{{}^{\circ}},\tau |D_{n-1}\right)\approx \text{Gam}(\tau |a,b)\cdot \prod_{k=1}^{K}\prod_{j=1}^{d_{k}}\prod_{r=1}^{R}N\left(u_{jr}^{k}|\mu_{jr}^{k},v_{jr}^{k}\right). \end{array}$$ (7)

Obviously, when *n* = 1, we have *a* = *a*_0_, *b* = *b*_0_, $\mu_{jr}^{k}=0$, and $v_{jr}^{k}=1$. Now, we can obtain the RHS of (6),

$$\begin{array}{c} p(U^{{}^{\circ}}|D_{n})\approx \text{Gam}(\tau |a,b)\cdot \prod_{k=1}^{K}\prod_{j=1}^{d_{k}}\prod_{r=1}^{R}N\left(u_{jr}^{k}|\mu_{jr}^{k},v_{jr}^{k}\right)\cdot N(y_{n}|1^{⊤}\left(u_{i_{n1}}^{1}{}^{\circ}\cdots {}^{\circ}u_{i_{nK}}^{K}\right),\tau^{-1}). \end{array}$$ (8)

Note that, only the factors that involve in the observed entry **i**_*n*_ will get their posterior updated. For other factors, their running posterior remains the same. We use the assumed density filtering framework. That is, we will project the RHS of (8) into the the exponentially family to obtain the approximation of $p(U^{{}^{\circ}},\tau |D_{n})$, which has the same factorized form in (7). Following Minka’s Ph.D. thesis, what we need to do is to compute the normalizer,

$$\begin{array}{c} Z=\int p\left(U^{{}^{\circ}},\tau |D_{n-1}\right)N(y_{n}|1^{⊤}\left(u_{i_{n1}}^{1}{}^{\circ}\cdots {}^{\circ}u_{i_{nK}}^{K}\right),\tau^{-1}), \end{array}$$ (9)

and then apply the following formula,

$$\begin{array}{c} \mu_{jr}^{k*}\leftarrow \mu_{jr}^{k}+v_{jr}^{k}\frac{\partial \;\text{log}\;Z}{\partial \mu_{jr}^{k}},\\ v_{jr}^{k*}\leftarrow v_{jr}^{k}-{\left(v_{jr}^{k}\right)}^{2}[{\left(\frac{\partial \;\text{log}\;Z}{\partial \mu_{jr}^{k}}\right)}^{2}-2\frac{\partial \;\text{log}\;Z}{\partial v_{jr}^{k}}]. \end{array}$$ (10)

Then we set the above as the updated mean and variance for the running posterior, namely,

$$\begin{array}{c} p\left(u_{jr}^{k}|D_{n}\right)=N\left(u_{jr}^{k}|\mu_{jr}^{k*},v_{jr}^{k*}\right). \end{array}$$ (11)

The posterior of *τ* are

$$\begin{array}{c} p\left(\tau |D_{n}\right)=\text{Gam}\left(\tau |a^{*},b^{*}\right). \end{array}$$ (12)

To obtain it, we need to compute the first and second moments of *τ*, with which to match a new Gamma distribution. Specifically, given

$$\begin{array}{c} \tilde{p}\left(\alpha ,\tau \right)\propto N\left(\alpha |m,\sigma^{2}\right)\text{Gam}\left(\tau |a,b\right)N\left(y_{n}|\alpha ,\tau^{-1}\right), \end{array}$$

we want to marginalize out *α*, and compute $E_{\tilde{p}}\left[\tau \right]$ and $E_{\tilde{p}}\left[\tau^{2}\right]$. Then according to

$$\begin{array}{c} \frac{a^{*}}{b^{*}}=E_{\tilde{p}}\left[\tau \right],\;\;\;\frac{a^{*}}{{b^{*}}^{2}}={\text{Var}}_{\tilde{p}}\left[\tau \right]=E_{\tilde{p}}\left[\tau^{2}\right]-{\left(E_{\tilde{p}}\left[\tau \right]\right)}^{2}, \end{array}$$

we can obtain

$$\begin{array}{c} a^{*}=\frac{{\left(E_{\tilde{p}}\left[\tau \right]\right)}^{2}}{{\text{Var}}_{\tilde{p}}\left[\tau \right]},\;\;\;b^{*}=\frac{E_{\tilde{p}}\left[\tau \right]}{{\text{Var}}_{\tilde{p}}\left[\tau \right]}. \end{array}$$ (13)

The normalizer (9), however, does not have a closed form. To address this problem, we use moment-match to construct a Gaussian posterior approximation of

$$\begin{array}{c} \alpha =1^{⊤}\left(u_{i_{n1}}^{1}{}^{\circ}\cdots {}^{\circ}u_{i_{nK}}^{K}\right). \end{array}$$ (14)

That is,

$$\begin{array}{c} p\left(\alpha |D_{n-1}\right)\approx N\left(\alpha |m,\sigma^{2}\right) \end{array}$$

where

$$\begin{array}{cc} m & =E_{p(U^{{}^{\circ}}|D_{n-1})}\left[\alpha \right]=E\left[1^{⊤}\left(u_{i_{n1}}^{1}{}^{\circ}\cdots {}^{\circ}u_{i_{nK}}^{K}\right)\right]\\ \sigma^{2} & =E_{p(U^{{}^{\circ}}|D_{n-1})}\left[\alpha^{2}\right]-m^{2}. \end{array}$$ (15)

E is the expectation operator. To compute the first-order and second-order moments, we define $a_{i}=u_{i_{n1}}^{1}{}^{\circ}\ldots {}^{\circ}u_{i_{nK}}^{K}$. First, we compute first-order moments:

$$\begin{array}{c} E_{p(U^{{}^{\circ}}|D_{n-1})}\left[\alpha \right]=E\left[1^{⊤}\left(u_{i_{n1}}^{1}{}^{\circ}\cdots {}^{\circ}u_{i_{nK}}^{K}\right)\right]. \end{array}$$ (16)

Now let us compute second-order moments.

$$\begin{array}{c} E_{p(U^{{}^{\circ}}|D_{n-1})}\left[\alpha^{2}\right]\\ =E\left[{\left(1^{⊤}a_{i}\right)}^{2}\right]=E\left[{\left(1^{⊤}\right)}^{2}{\left(a_{i}\right)}^{2}\right]\\ =E\left[1^{⊤}a_{i}\cdot a_{i}^{⊤}1\right]=E\left[tr\left(a_{i}^{⊤}1\cdot 1^{⊤}a_{i}\right)\right]\\ =E\left[tr\left(11^{⊤}a_{i}a_{i}^{⊤}\right)\right]=tr\left(11^{⊤}\cdot E\left[a_{i}a_{i}^{⊤}\right]\right)\\ =tr(11^{⊤}\cdot E\left[\left(u_{i_{n1}}^{1}{}^{\circ}\cdots {}^{\circ}u_{i_{nK}}^{K}\right)\cdot \left(u_{i_{n1}}^{1}{}^{\circ}\cdots {}^{\circ}u_{i_{nK}}^{K}\right)\right]\\ =tr(11^{⊤}\cdot E\left[\left(u_{i_{n1}}^{1}\cdot {\left(u_{i_{n1}}^{1}\right)}^{⊤}\right)\right]{}^{\circ}\cdots {}^{\circ}E\left[u_{i_{nK}}^{K}\cdot {\left(u_{i_{nK}}^{K}\right)}^{⊤}\right]) \end{array}$$ (17)

Where *tr* performs trace operation.

Let’s first fix *τ*, marginalize the Gamma function, the normalizer (9) turn into

$$\begin{array}{cc} Z & =\int p\left(\alpha |D_{n-1}\right)N\left(y_{n}|\alpha ,\tau^{-1}\right)d\alpha \\ & \approx \int N\left(\alpha |m,\sigma^{2}\right)N\left(y_{n}|\alpha ,\tau^{-1}\right)d\alpha \\ & =\int N(y_{n}|m,\sigma^{2}+\tau^{-1})d\tau \end{array}$$ (18)

Since the marginal distribution of *y*_*n*_ is equal to the above Eq (18) and, at the same time, because the marginal distribution of *y*_*n*_ is equal to the integral of the marginal probability density function of *y*_*n*_, we obtain

$$\begin{array}{cc} Z & \approx \int N\left(\alpha |m,\sigma^{2}\right)N\left(y_{n}|\alpha ,\tau^{-1}\right)d\alpha \\ & =N(y_{n}|m,\sigma^{2}+\tau^{-1}). \end{array}$$ (19)

In this way, we could update the posterior in a simple and efficient fashion without using the complex form of KL-Divergence (Kullback-Leibler Divergence) minimization in variational inference framework [37] like POST [6]. The online Bayesian streaming structure method of fixing *τ* (BS-CP1) is shown in Algorithm 1.

**Algorithm 1** Streaming algorithm BS-CP1

1: **while** A new set of tensor entries $D_{n}$ arrived, **do**

2:  For each brand-new object *n* at each mode *k*, initialize $q\left(u_{jr}^{k}\right)=N\left(u_{jr}^{k}|0,1\right)$.

3:  **repeat**

4:   Update each $q^{*}\left(u_{jr}^{k}\right)$ associated with $D_{n}$, *i*.*e*., *j* ∈ [1, *d*_*k*_], *r* ∈ [1, *R*] and *k* ∈ [1, *K*], first update Z using (19), then use Eqs (10), (11) and (15).

5:  **until** Convergence has been reached, or the maximum number of iterations have finished.

6:  Set each $q\left(u_{jr}^{k}\right)=q^{*}\left(u_{jr}^{k}\right)$ for 1 ≤ *j* ≤ *d*_*k*_, 1 ≤ *k* ≤ *K*.

7: **end while**

8: **return** The posteriors of the embedding vectors $q\left(u_{jr}^{k}\right)=N\left(u_{jr}^{k}|\mu_{jr}^{k*},v_{jr}^{k*}\right)$ for each object that have been seen, 1 ≤ *k* ≤ *K*.

### Gauss-Laguerre quadrature method

Now, lets consider take Gamma function into consideration. To compute the normalizer (9), we have

$$\begin{array}{cc} Z & =\int p\left(\alpha |D_{n-1}\right)p\left(\tau |D_{n-1}\right)N\left(y_{n}|\alpha ,\tau^{-1}\right)d\alpha d\tau \\ & \approx \int N\left(\alpha |m,\sigma^{2}\right)\text{Gam}\left(\tau |a,b\right)N\left(y_{n}|\alpha ,\tau^{-1}\right)d\alpha d\tau \\ & =\int N\left(y_{n}|m,\sigma^{2}+\tau^{-1}\right)\text{Gam}\left(\tau |a,b\right)d\tau . \end{array}$$ (20)

Even though we use moment-match, the above Eq (20) still can be a bit tricky to get. It involves integration that contains exponential terms, requiring complex computation. Gauss-Laguerre quadrature [13] is a Gaussian integration method for numerical integration, which is a generalization of the Gaussian integration method and is specifically used to compute integrals over semi-infinite intervals [0, +∞). This integration method uses the orthogonality of Laguerre polynomials to improve the accuracy and efficiency of integration. For the above reasons, we use Gauss-Laguerre quadrature to accelerate the convergence speed and improve the calculation accuracy. Let’s transform the above equation into

$$\begin{array}{cc} Z & \approx \int N\left(y_{n}|m,\sigma^{2}+\tau^{-1}\right)\frac{b^{a}}{\Gamma (a)}\tau^{a-1}e^{-b\tau }d\tau \\ & =\frac{1}{\Gamma (a)}\int_{0}^{\infty }N\left(y_{n}|m,\sigma^{2}+b/s\right)s^{a-1}e^{-s}ds\;\;\;\left(s=b\tau \right). \end{array}$$ (21)

we can use Gauss-Laguerre quadrature to compute (21),

$$\begin{array}{c} \int_{0}^{\infty }f\left(s\right)e^{-s}ds\;\approx \sum_{j=1}^{T}w_{j}f\left(s_{j}\right). \end{array}$$ (22)

where *w*_*j*_ and *s*_*j*_ are quadrature weights and nodes. Here

$$\begin{array}{c} f\left(s\right)=N\left(y_{n}|m,\sigma^{2}+b/s\right)s^{a-1}. \end{array}$$

This leads to two problems: the selection of nodes and the setting of corresponding weights. How to choose the nodes and weights of the Gauss-Laguerre function is tricky. We can choose the value of node n through Laguerre’s formula [13], but this method is not always optimal, we need to try. The good news is that engineers have given the commonly used Gauss-Laguerre quadrature nodes and quadrature weights [38]. We sample from the smallest n = 1 until n = 5. The larger n, the higher the accuracy, but if *f*(*s*) is not very complicated, such as our *f*(*s*), the larger n will result in more computation and almost no improvement in accuracy.

To ensure numerical stability, we observe

$$\begin{array}{c} \;\text{log}\;Z\approx -\;\text{log}\;\Gamma \left(a\right)+\;\text{log}\;[\sum_{j=1}^{T}\text{exp}(\;\text{log}\;w_{j}+\;\text{log}\;f\left(s_{j}\right))]. \end{array}$$ (23)

Now, we can compute Z in an easy way.

Back to $p\left(\tau |D_{n}\right)=\text{Gam}\left(\tau |a^{*},b^{*}\right)$. We also need to obtain the moments $E_{\tilde{p}}\left[\tau \right]$ and $E_{\tilde{p}}\left[\tau^{2}\right]$ To obtain them, all we need to do is to run GL quadrature twice more,

$$\begin{array}{cc} Z_{1} & =\int_{0}^{\infty }\tau \cdot N\left(y_{n}|m,\sigma^{2}+\tau^{-1}\right)\text{Gam}\left(\tau |a,b\right)d\tau \\ Z_{2} & =\int_{0}^{\infty }\tau^{2}\cdot N\left(y_{n}|m,\sigma^{2}+\tau^{-1}\right)\text{Gam}\left(\tau |a,b\right)d\tau , \end{array}$$ (24)

which using the same variable change, we obtain

$$\begin{array}{cc} Z_{1} & =\frac{1}{\Gamma (a)b}\int_{0}^{\infty }N\left(y_{n}|m,\sigma^{2}+b/s\right)s^{a}e^{-s}ds\\ Z_{2} & =\frac{1}{\Gamma \left(a\right)b^{2}}\int_{0}^{\infty }N\left(y_{n}|m,\sigma^{2}+b/s\right)s^{a+1}e^{-s}ds. \end{array}$$ (25)

We can use the same computational trick above to compute $\;\text{log}\;Z_{1}$ and $\;\text{log}\;Z_{2}$, and get

$$\begin{array}{cc} & E_{\tilde{p}}\left[\tau \right]=\text{exp}\left(\;\text{log}\;Z_{1}-\;\text{log}\;Z\right),\;\;\;\;\;\\ & E_{\tilde{p}}\left[\tau^{2}\right]=\text{exp}\left(\;\text{log}\;Z_{2}-\;\text{log}\;Z\right). \end{array}$$ (26)

Now, we can use (11) to update the posterior and the factors associated with the entry.

By using Gauss-Laguerre quadrature method, the accuracy is improved, and the model converges more quickly, it is more efficient and reliable, and a further improvement on our original method. The Gauss-Laguerre quadrature fasioned online Bayesian streaming structure (BS-CP2) is summarized in Algorithm Algorithm 2.

Our algorithm is a model-based recommendation algorithm that tries to quantify how much a user likes an item they haven’t encountered before. To achieve this, we use tensor decomposition and Bayesian posterior probability inference to train a vector of items (for a specific user) and then build a model to predict the user’s score for a new item. The principle of CP tensor decomposition algorithm is clear and clear, and the Bayesian probability inference model is often used in daily life, thus our BS-CP is easy to explain to users and has good interpretability.

**Algorithm 2** Streaming algorithm BS-CP2

1: Initialize *p*(*τ*) with an uninformative Gamma distribution, *p*(*τ*) = *Gam*(*τ*|10^−3^, 10^−3^).

2: **while** A new set of tensor entries $D_{n}$ arrived, **do**

3:  For each brand-new object *n* at each mode *k*, initialize $q\left(u_{jr}^{k}\right)=N\left(u_{jr}^{k}|0,1\right)$.

4:  **repeat**

5:   Update each $q^{*}\left(u_{jr}^{k}\right)$ associated with $D_{n}$, *i*.*e*., *j* ∈ [1, *d*_*k*_], *r* ∈ [1, *R*] and *k* ∈ [1, *K*], first update Z using (21), (22), (23), then use Eqs (10), (11) and (15).

6:   Update q*(*τ*) using (12), (13), (26).

7:  **until** Convergence has been reached, or the maximum number of iterations have finished.

8:  Set each $q\left(u_{jr}^{k}\right)=q^{*}\left(u_{jr}^{k}\right)$ for 1 ≤ *j* ≤ *d*_*k*_, 1 ≤ *k* ≤ *K*. Set *q*(*τ*) = q*(*τ*).

9: **end while**

10: **return** The posteriors of the embedding vectors $q\left(u_{jr}^{k}\right)=N\left(u_{jr}^{k}|\mu_{jr}^{k*},v_{jr}^{k*}\right)$ for each object that have been seen, 1 ≤ *k* ≤ *K*.

## Complexity

The time complexity of BS-CP1 in each batch is $O(Kr^{3})$, where r is the rank, space complexity is $O(\sum_{k=1}^{K}d_{k}r^{3}+K)$, which is to store the posterior mean and covariance of the latent factors. The time complexity of BS-CP2 is $O(nKr^{3})$, where n is the nodes of Gauss-Laguerre quadrature method, the number of nodes n of the Gauss-Laguerre quadrature is fixed in each update step. Space complexity is $O(\sum_{k=1}^{K}d_{k}nr^{2}+K)$, which is to store the embedding posteriors, the data batch, the nodes of Gauss-Laguerre quadrature method, and the posterior *q*(*τ*). It can be concluded that the time complexity of BS-CP2 is worse than BS-CP1 but the space complexity is better due to the Gauss-Laguerre quadrature integral value approximate method.

## Experiment

### Model comparison

The method we chose for comparison is the latest algorithm with a structure similar to our method, it does not include some non-universal methods generated for a certain situation or focus on solving a certain class of problems (such as time parameters participating in tensor decomposition or under sparse data) used in different scenarios. Our method based on Bayesian streaming data processing, which can be used in most application scenarios (except extreme conditions). Therefore, we also choose the latest SOTA methods that can be applied to most scenarios for comparison. We compare our approach to the following three categories of approaches:

1. the state-of-the-art (SOTA) multilinear streaming tensor decomposition algorithm:
  - POST [6], which was based on SVB, using CP decomposition.
  - BASS-Tucker [14], using Tucker decomposition, placed a spike-and-slab prior over the core tensor elements, build the model based on ADF and moment-match.
2. SOTA static multilinear decomposition methods:
  CP-WOPT [5], this is a CP decomposition by conjugate gradient descent method.
  CP-ALS [16], CP decomposition via alternating least square updates, which has been used widely.
  Tucker-ALS [4], Tucker decomposition via alternating least square updates.
3. SOTA continuous-time multilinear tensor decomposition algorithm:
  CT-CP [15],in this method, the time tensor value function is used to integrate time and context information, and the time-varying coefficient model of time tensor decomposition is established by polynomial spline approximation.

### Datasets

We evaluated BS-CP1 and BS-CP2 in four real-world applications. The information of our dataset is summarized in Table 2.

1. Beijing-15k [39], the dataset includes six pollutants from 12 state-controlled air quality monitoring sites from 2013 to 2017. Air quality data were obtained from the Beijing Municipal Environmental Monitoring Center. We randomly selected 15K continuous measurements and their time stamps for training and testing. Its a real-valued four-way tensor of size 12 × 6 × 12 × 1461.
2. Beijing-20k [39],Compared with Beijing-15k, we reduce the acquisition of one factor and simplify the data set. For training and testing, we randomly selected 20K continuous measurements. Its a real-valued three-way tensor of size 12 × 6 × 315.
3. MovieLens-1m [40], a two-mode continuous tensor of size 6040 × 3706, consisting of (user, movie) ratings. The number of observed elements is 1000,209.
4. Fit Record [41], an EndoMondo user’s exercise log of outdoor activities. We extracted three-way interactions between 500 users, 20 exercise types, 50 attitudes. That is 500 × 20 × 50 × 101. The result is the user’s heart rate during exercise, measured for 4,000 to 6,000 seconds. We collected 49,978 heart rates and time stamps in total.

**Table 2:** Information of four datasets.

datasets	dimension	size	items	elements	sparsity
**Beijing-15k**	4	12 × 6 × 12 × 1461	(districts, pollution, hour, concentration)	15000	3%
**Beijing-20k**	3	12 × 6 × 315	(districts,pollution,concentration)	20000	3%
**MovieLens-1m**	2	6040 × 3706	(user, movie)	1000,209	95.81%
**Fit Record**	4	500 × 20 × 50 × 101	(user, sport type, altitude)	49,978	99.9094%

### Settings

We implemented BS-CP1 and BS-CP2 using software environment: Pytorch [42], Windows, hardware environment: Intel(R) Core(TM) i9–14900HX 2.20GHz, 16G RAM, NVIDIA GeForce RTX 4060 GPU.

We first set *a* = 10^−3^, *b* = 10^−3^, $\mu_{jr}^{k}$ a random number ranging from 0 to 1, and $v_{jr}^{k}=1$. We used the original implementation of all the comparison methods and their default settings (such as the maximum number of iterations, etc.). As mentioned in the previous algorithm, we fixed tau to 1 in the BS-CP1 method, both BS-CP1 and BS-CP2 are set according to the initialize parameter in algorithm BS-CP1 and Algorithm BS-CP2 respectively.

We evaluate the final predictive performance when all stream entries have been processed. To this end, 15,000 observations of Beijing-15k datasets were randomly divided into 75 percent for training and 25 percent for testing, and 20,000 observations of Beijing-20k datasets were randomly divided into 4 for training and testing: Due to the large MovieLens-1M datasets, 90 percent of MovieLens-1M’s 1,000,209 data are used for training and 10 percent for testing, and 49,978 observation items of Fit Record dataset are randomly divided into 4:1 training set and test set ratio. For static tensor decomposition, iterative static decomposition requires repeated access to the entire data. We fixed the batch as 1, repeated the experiment 5 times, and calculated the mean root mean square error (RMSE) of the real-valued dataset. We take the final prediction performance of each method when the tensor decomposition rank R is 3,5,8,10, respectively. Then, we give the total time for BS-CP1 and BS-CP2 to complete a round under different ratios of training set and test set (0.25 and 0.1) when the values of R are different (R = 3,5,8,10). In addition, we explored the relationship between the selection of Gauss-Laguerre integral nodes and RMSE. We selected nodes n = 1,2,3,4,5, and the corresponding weights (obtained from the Gauss-Laguerre quadrature nodes and quadrature weights Table [38]). Such a setup evaluates the relationship between the rank of the tensor decomposition and the accuracy of the calculation.

For all algorithms for which experiments were performed, we used the original implementations of all competing methods and their default Settings (for example, the maximum number of iterations for static decomposition). We evaluate the final prediction performance, that is, when all stream entries have been processed. For POST, MASS-Tucker, CT-CP and our BS-CP1 and BS-CP2, we randomly divide the training entries into mini-batch streams. Other approaches perform iterative static decomposition, requiring repeated access to the entire data.

### Metric

We selected RMSE (Root Mean Squared Error) as the evaluation metric. RMSE is a commonly used performance metric for machine learning, it is the square root of MSE (Mean Squared Error), which can reflect the average difference between the predicted value and the true value, and its calculation formula is:

$$\begin{array}{c} RMSE=\sqrt{\sum_{i=1}^{n}{\left(Y_{i}-\hat{Y_{i}}\right)}^{2}/n} \end{array}$$ (27)

where *n* is the number of samples, *Y*_*i*_ is the *i*th true value, $\hat{Y_{i}}$ is the *i*th predicted value. RMSE can reflect the standard deviation of the real error and facilitate error analysis and is more sensitive to outliers.

Meanwhile, we included an examination of the standard deviation (STD) of our RMSE to indicate the stability of our model. The formula used to calculate standard deviation is as follows:

$$\begin{array}{c} STD=\sqrt{\sum_{j=1}^{a}{\left(x_{j}-\mu \right)}^{2}/a} \end{array}$$ (28)

where *a* is the number of runs of the algorithm, *x*_*j*_ is the results obtained for each run of the algorithm, *μ* is the average of the results from each run of the algorithm (the sum of the algorithm results divided by *a*). The value of the standard deviation is a very effective indication of the stability of the algorithm.

## Results

### RMSE of different methods

We show the line plots of RMSE values and standard deviations(STD) of all comparison methods and our two methods when R is 3,5,8,10 in S5 Fig. It can be seen from S5 Fig that, except slightly worse than POST in some R values in Beijing-20k dataset and worse than CT-CP when R = 10 in Fit Record dataset, BS-CP1 and BS-CP2 are better than the comparison method in all the other cases. This demonstrates the superiority of the structure of our BS-CP algorithm.

However, observing the performance of POST on the four datasets, it can be seen that the performance of POST algorithm on different datasets is different. This may be due to the fact that POST algorithm uses SVB to approximate Bayesian inference and falls into local optimal solutions. As a result, it is sensitive to some datasets (Beijing-20k) and insensitive to others (MovieLens-1m). However, our method performs well on all data sets, which shows that our method has certain universality. However, the reason why CT-CP outperforms our method when R = 10 May be because the Fit Record dataset itself is sensitive to temporal information, and CT-CP method embeds temporal information into tensor decomposition, which has advantages in time prediction. Although our method does not focus on the time factor, the effect is not worse than CT-CP which specifically builds the time-varying coefficient model of time tensor decomposition, which is enough to show the wide application and excellent effect of our method. Meanwhile, in all cases, BS-CP1 and BS-CP2 are better than SOTA static decomposition method.

More important, it can be seen from S5(c) and S5(d) Fig that our BS-CP structure algorithm has a larger improvement than most other methods. We put the RMSE values of all the streaming data processing methods together for comparison, which are presented in S6 Fig. As it can be seen from the S6 Fig, the red and purple columns on the right side (BS-CP1 and BS-CP2) have better RMSE than most of the columns on the left side for streaming methods used for comparison. Looking at the last two datasets, comparing our method BS-CP1 with the SOTA streaming method BASS-Tucker, we achieve 31.8% and 33.3% RMSE improvement in these two datasets. Consider that MovieLens-1m and Fit Record are relatively large datasets (1000,209 and 49,978 elements), such results further illustrate the effect of our method when facing large real datasets.

And we can see from the numerical figures in Table 3, BS-CP is superior to the comparison method in most cases, among them, MovieLens-1m has the most obvious improvement. At the same time, it can be seen that in Table 3, the STD value of our BS-CP methods are smaller than that of most methods, which shows that the stability of the BS-CP method is generally better than that of other methods. At the same time, the STD value of BS-CP2 algorithm is smaller than that of BS-CP1, indicating the advantage of using Gauss-Laguerre quadrature method. The results of these demonstrations prove the advantage of BS-CP in prediction accuracy.

**Table 3:** Prediction error results averaged from five runs. These are final prediction error with R = 3, BS-CP1 and BS-CP2 run with R = 3 and nodes n = 5.

	RMSE	Beijing-15k	Beijing-20k	MovieLens-1m	Fit Record
**static**	CP-ALS	0.867±0.009	0.567±0.022	0.927±0.002	0.905±0.009
	CP-WOPT	1.002±0.190	0.859±0.529	0.816±0.001	0.882±0.049
	Tucker-ALS	0.838±0.026	0.600±0.022	0.914±0.001	0.846±0.006
**stream**	POST	0.678±0.028	**0.511**±**0.001**	1.601±0.010	0.660±0.012
	BASS-Tucker	0.937±0.025	1.085±0.030	0.913±0.001	0.981±0.021
**time**	CT-CP	0.897±0.012	0.625±0.014	1.023±0.002	0.679±0.009
**ours**	BS-CP1	0.546±0.012	0.529±0.010	**0.622**±**0.002**	**0.654**±**0.015**
	BS-CP2	**0.536**±**0.011**	0.529±0.029	0.629±0.001	0.659±0.011

### Stability and saliency of the model

Table 3 and S5 Fig also reflect the effect of different data set types on the value of the standard deviation. In S5 Fig, we can see that the standard deviation of the larger data sets (MovieLens-1m and Fit Record) is smaller, because the stability of the algorithm is improved with more training data. The largest dataset, MovieLens-1m, has the smallest standard deviation, meaning it has the best stability. Meanwhile, by comparing the STD values of our methods BS-CP1 and BS-CP2 with those of other methods, it can be seen from S5 Fig that the STD values of our method are better than those of the other compared methods, indicating that our methods has good stability.

In order to prove the significance of the advantages of our method, BS-CP1 and BS-CP2 are compared with other comparison methods by student’s t test. Follow the student’s t test formula:

$$\begin{array}{c} t=\frac{\bar{x_{1}}-\bar{x_{2}}}{\sqrt{{S_{1}}^{2}/N_{1}+{S_{2}}^{2}/N_{2}}} \end{array}$$ (29)

where $\bar{x_{1}}$ and $\bar{x_{2}}$ are the average of the first and second set of data, ${S_{1}}^{2}$ and ${S_{2}}^{2}$ are the STD of them, *N*_1_ and *N*_2_ are the number of first and second set of data.

In this way we can easily obtain the t test value of each compared method compared with BS-CP1 and BS-CP2 within all datasets. By looking up the table of critical values for the t-distribution we can obtain the range of p-values corresponding to the t values. The conclusion we have is that except POST, BS-CP1 and BS-CP2 shows an significant improvement (*p* < 0.05) compared to all other comparison methods, demonstrates the saliency and effectiveness of our approach.

### Training time

In S7 Fig, we show the running time of BS-CP1 and BS-CP2 under different datasets when *R* = (3, 5, 8, 10). We can intuitively see from S7 Fig that BS-CP2 takes more time than BS-CP1, which is the disadvantage of BS-CP2’s higher stability. It can be seen that the overall training time of BS-CP2 shows a downward trend, same cases in BS-CP1 when the datasets are Beijing-15k and MovieLens-1m. This phenomenon may be attributed to the application of the Gauss-Laguerre quadrature method in BS-CP2, which is based on the orthogonality of the Laguerre polynomial and helps to simplify the calculation process of the integral. It is not constrained by the value of R. On the contrary, in the more complicated case of exponential integration, The Gauss-Laguerre quadrature method may converge faster. This further indicates the advantage of the BS-CP2 method. In Beijing-20k and Fit Record datasets, BS-CP1 showed an up-and-down phenomenon with an increasing or not obvious trend. On the whole, the algorithm time of BS-CP1 and BS-CP2 shows a trend of decreasing with the increase of R. This shows that our method has some scalability, it also shows that the scalability of BS-CP2 is better than that of BS-CP1.

S8 Fig shows the time consuming of BS-CP2 algorithm when the number of nodes n takes different values. We can see that the smaller n is, the shorter the training time, and the training time is proportional to the value of n. It shows that BS-CP2 algorithm has good linear scalability.

### Effect of the number of nodes on RMSE

We showed the relationship between RMSE and node values n in S9 Fig. As can be seen from it, RMSE value is not simply a monotone function with node n value. In S9(a) Fig, RMSE value increases slightly (greater than 0.001) as the number of nodes increases. As the increase is too small (less than 0.0001) in S9(b) Fig, we drew the RMSE value only when R = 3 for comparison, and it can be seen that the RMSE value slightly increases with the number of nodes. S9(c) Fig is the same as above, RMSE increases as the value of node n increases. But in S9(d) Fig, NAN value occurs when r = 3, n = 1, but when r = 8, n = 1, RMSE is preferable to the minimum value 0.529605, which is much better than rest of the methods. The next best case without a NAN value where the value of a node is n = 5.

This is because the Gauss-Laguerre quadrature has the property of fast convergence. Although increasing the number of nodes means increasing the training time, the accuracy of integration will improve rapidly with the increase of the number of nodes and weights.

## Conclusion

A streaming Bayes CP tensor decomposition algorithm BS-CP is proposed. This method can effectively process streaming data and provide a posterior update. On the basis of this framework, we propose two algorithms, BS-CP1 and BS-CP2, in which BS-CP1 is a simple implementation method of BS-CP, and BS-CP2 uses Gauss-Laguerre function to reduce the error of posterior update parameters and further improve the accuracy of the algorithm. We compare these two algorithms with static and streaming SOTA tensor decomposition recommended algorithms, and select four real world data sets of different sizes for validation. By comparing their RMSE values, the RMSE values of our two algorithms are overwhelmingly superior to those of the selected method, and compared with the advanced streaming SOTA method, the RMSE values of our two algorithms are better than those of the selected method. Greater improvements were achieved on large data sets, which demonstrated the advantages of our approach.

In addition, we explored the actual training time of BS-CP1 and BS-CP2 methods and the relationship between time and the selection of R-value. As a whole, BS-CP2 shows a trend of decreasing algorithm time with the increase of R value, while BS-CP1 shows a fluctuation pattern. At the same time, the influence of the selection of BS-CP2 node value n on the time consuming is also explored. Then, we explored the influence of the selection of node value n of BS-CP2 on RMSE. Except for some abrupt points in the last dataset, RMSE value mostly increased with the increase of n value. These experiments prove the stability and scalability of our algorithm.

However, our method also has certain limitations, if the data contains many zeros or missing values, CP decomposition may not accurately capture the data structure, and additional techniques (such as regularization or low-rank approximation) are required to handle sparse data. In non-random noise environment, CP decomposition may be affected by noise, resulting in inaccurate decomposition results. If the Moment Match method does not select enough moments or reasonable moments, it may be affected by noise, resulting in inaccurate approximate probability density function. The accuracy of the Gauss-Laguerre quadrature method may be compromised in high-noise environments, especially when the distribution of noise is unknown or does not match the type of integral function. In the next step, we will improve our method for extreme environments (sparse data or high-noise environments), such as regularization, low-rank approximation or smoothing of the data, so that our algorithm can be applied more widely. At the same time, our algorithm does not consider the importance of time variables. In the next step, time parameters will be introduced into tensor decomposition to make our algorithm more suitable for time-sensitive data.

## Supporting information

S1 Fig CP decomposition and Tucker decomposition when the tensor is three mode. (TIF)

S2 Fig The structure diagram of the BS-CP algorithm. (TIF)

S3 Fig Online Bayesian Streaming Structure. (TIF)

S4 Fig CP decomposition Bayes network. (TIF)

S5 Fig Predictive performance with different numbers of factors. The results are averaged over 5 runs. The performance of several baselines are incomplete, because they are far worse than all the other methods and hence not included.
(ZIP)

S6 Fig Streaming method RMSE compared to BS-CP1 and BS-CP2. (TIF)

S7 Fig The total time taken to complete the data set once, when BS-CP1 and BS-CP2 are at different values of R= (3,5,8,10), n = 5. (TIF)

S8 Fig The relationship between the number of nodes and the time consuming of the BS-CP2 algorithm is shown here as dataset Beijing-15k, when R = 3,5,8,10. (TIF)

S9 Fig When different node values n (1, 2, 3, 4, 5), the relationship between RMSE and n values of the four above datasets is presented in (a),(b),(c),(d) when *R* values are different. Note that some points have no value because the value is NAN. (b) and (c) are RMSE when R = 3 because the difference between the RMSE values of the two sets of data is too small, we chose an obvious drawing way.
(TIF)
