# Supplementary figures and images for: BS-CP: Efficient streaming Bayesian tensor decomposition method via assumed density filtering
Source: PLoS One. 2024 Dec 2;19(12):e0312723. doi: 10.1371/journal.pone.0312723 (PMC11611110; doi:10.1371/journal.pone.0312723)

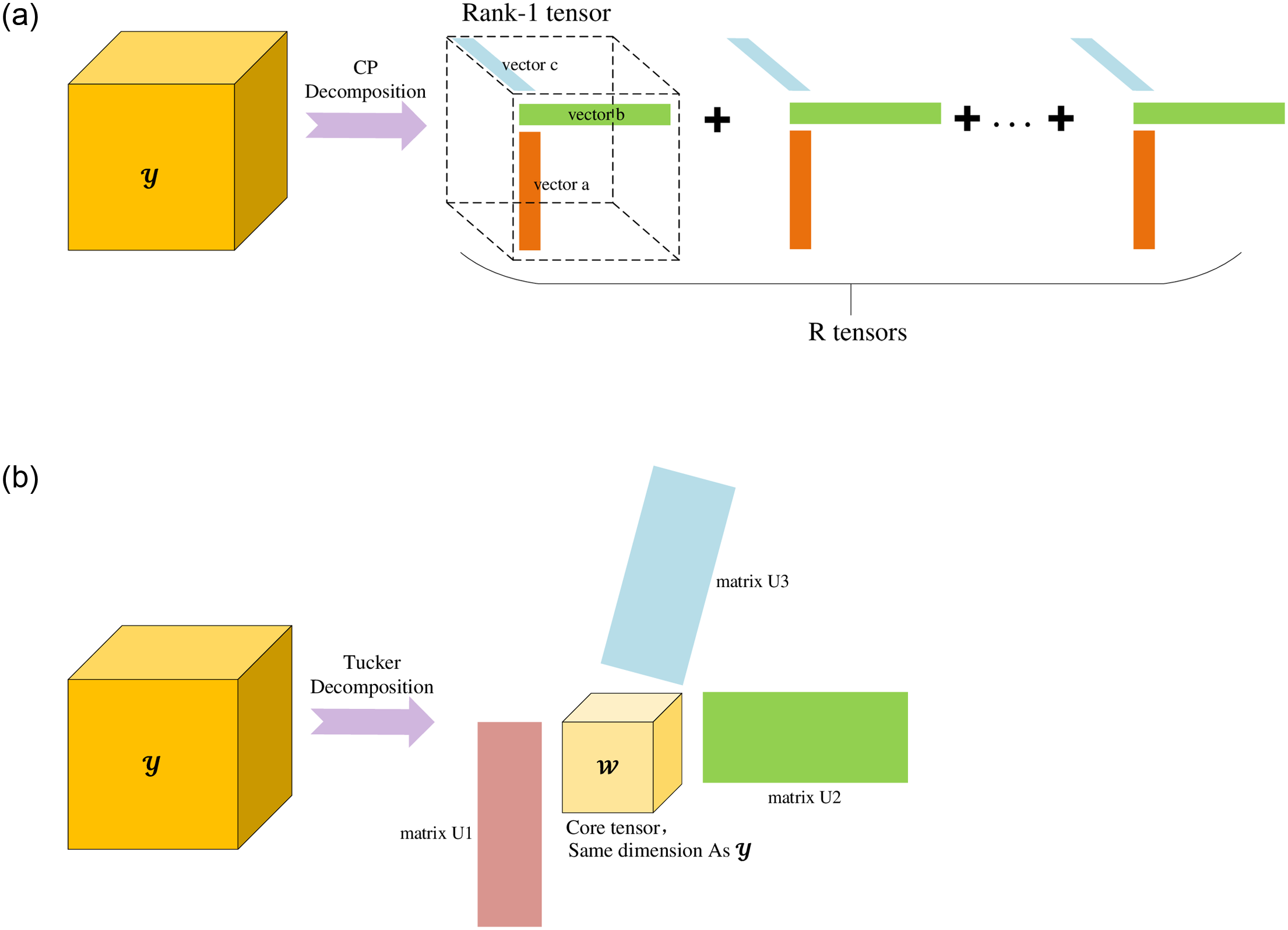

Supplement: S1 Fig — (TIF) [file pone.0312723.s001.tif]

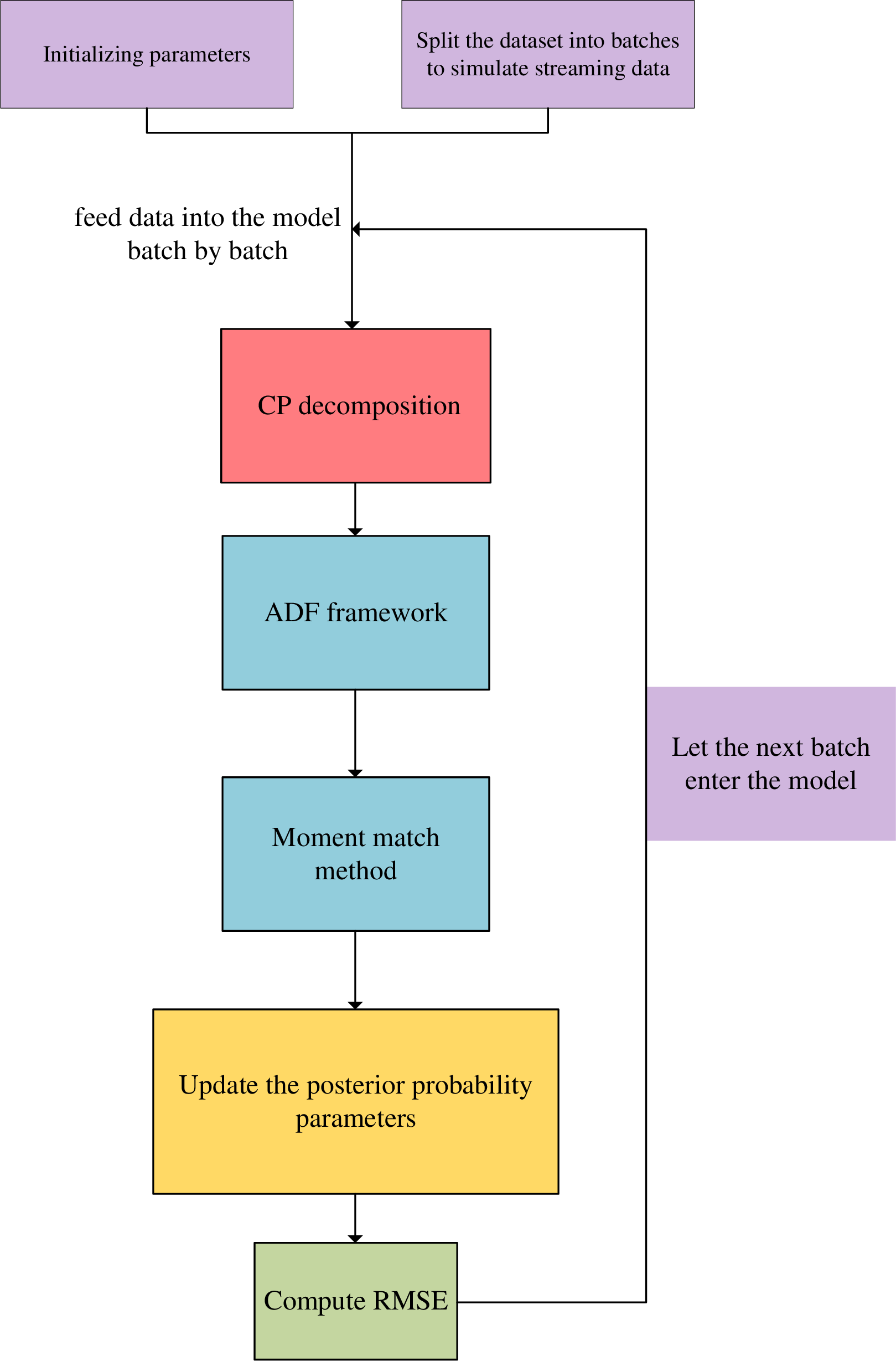

Supplement: S2 Fig — (TIF) [file pone.0312723.s002.tif]

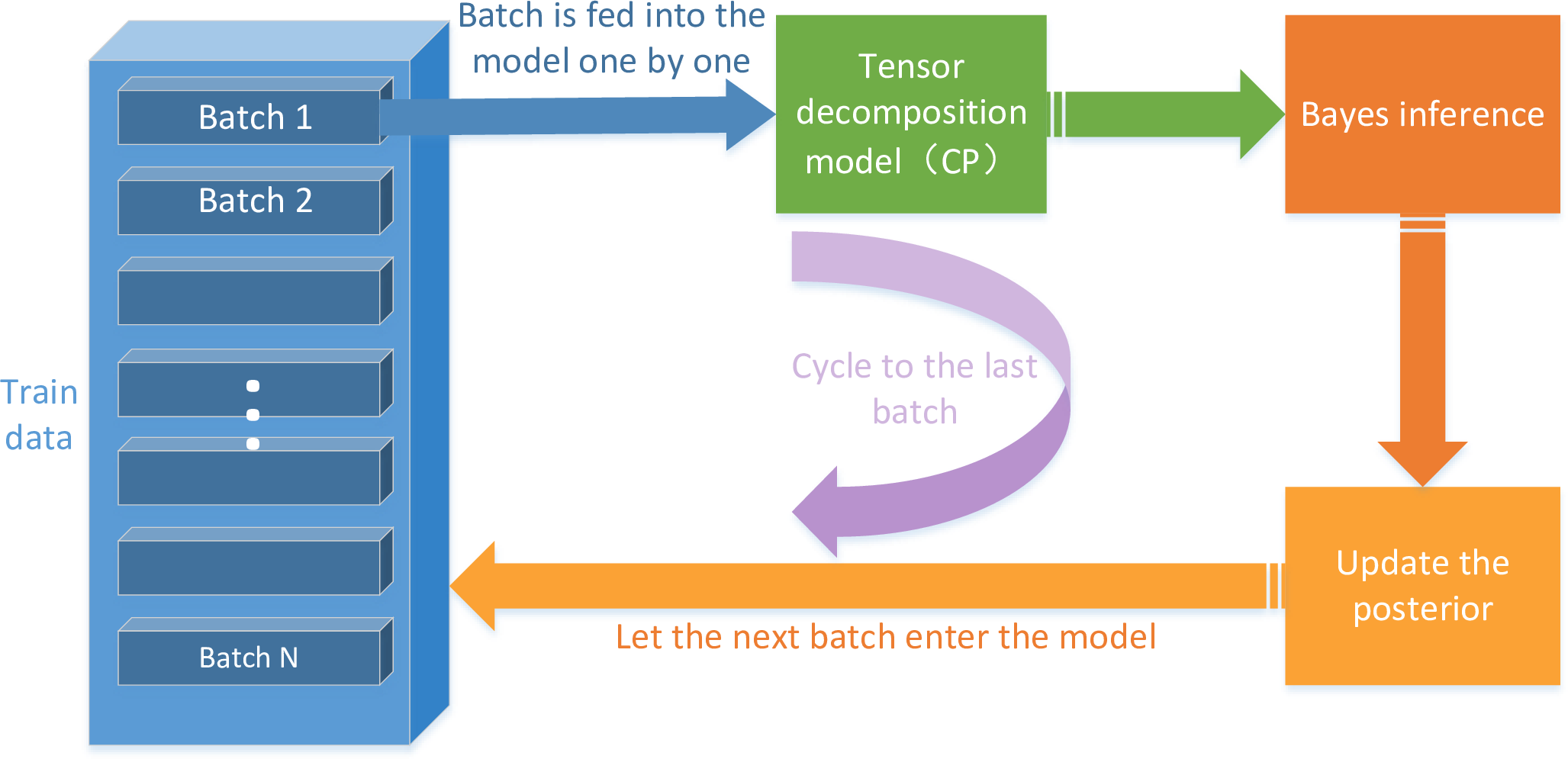

Supplement: S3 Fig — (TIF) [file pone.0312723.s003.tif]

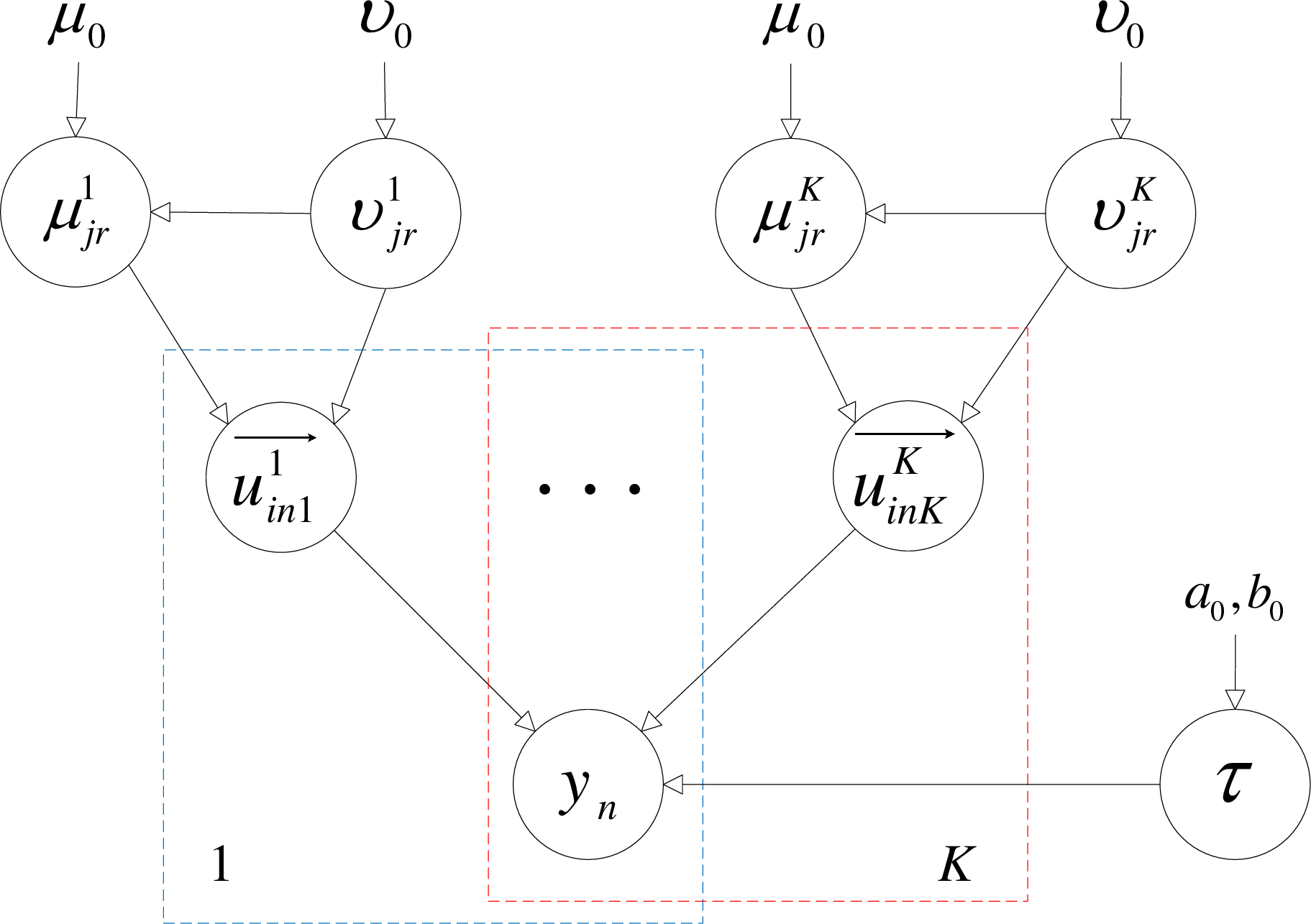

Supplement: S4 Fig — (TIF) [file pone.0312723.s004.tif]

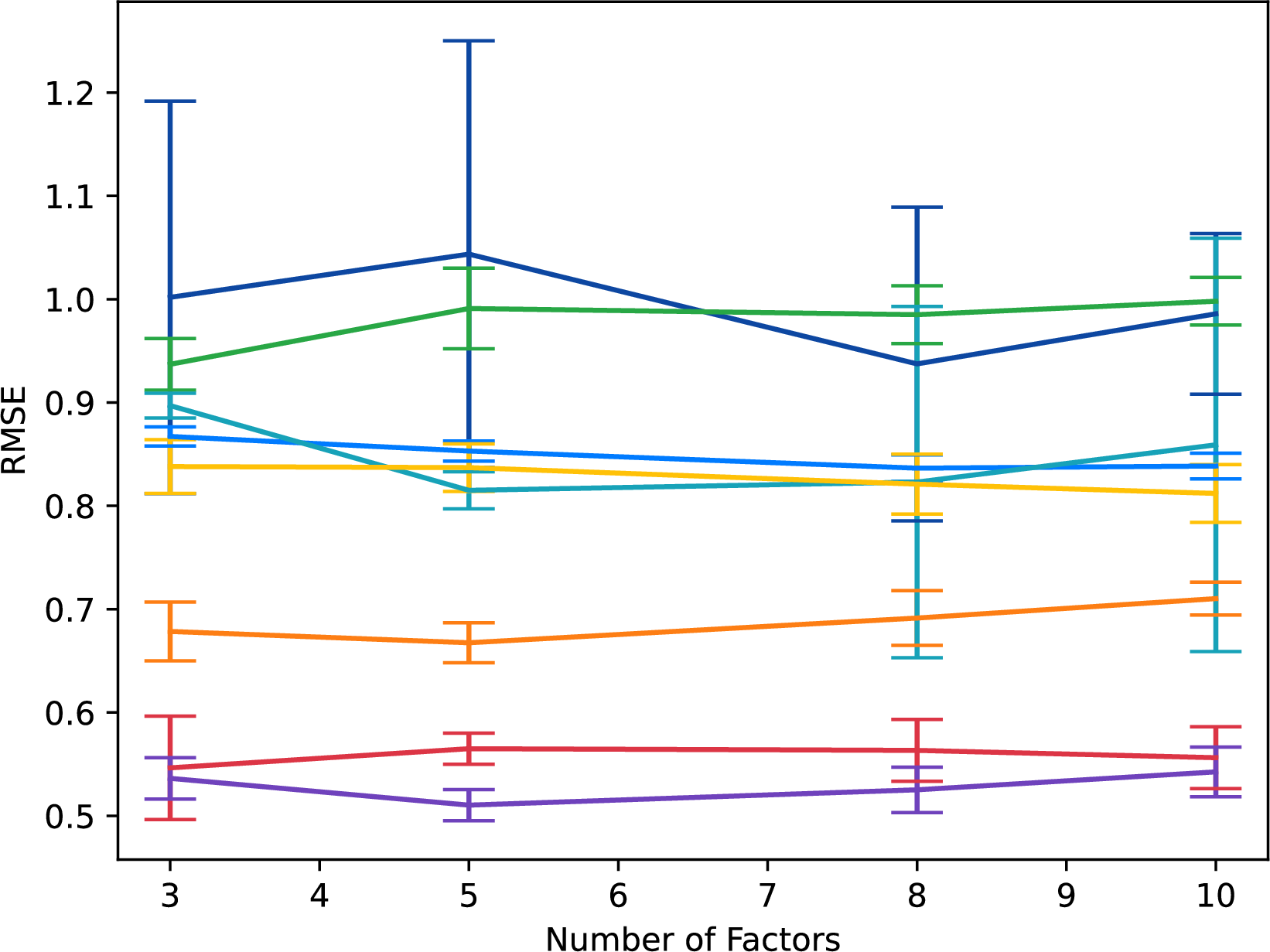

Supplement: S5 Fig — The results are averaged over 5 runs. The performance of several baselines are incomplete, because they are far worse than all the other methods and hence not included. (ZIP) [file pone.0312723.s005.zip › S5 Fig.(a).tif]

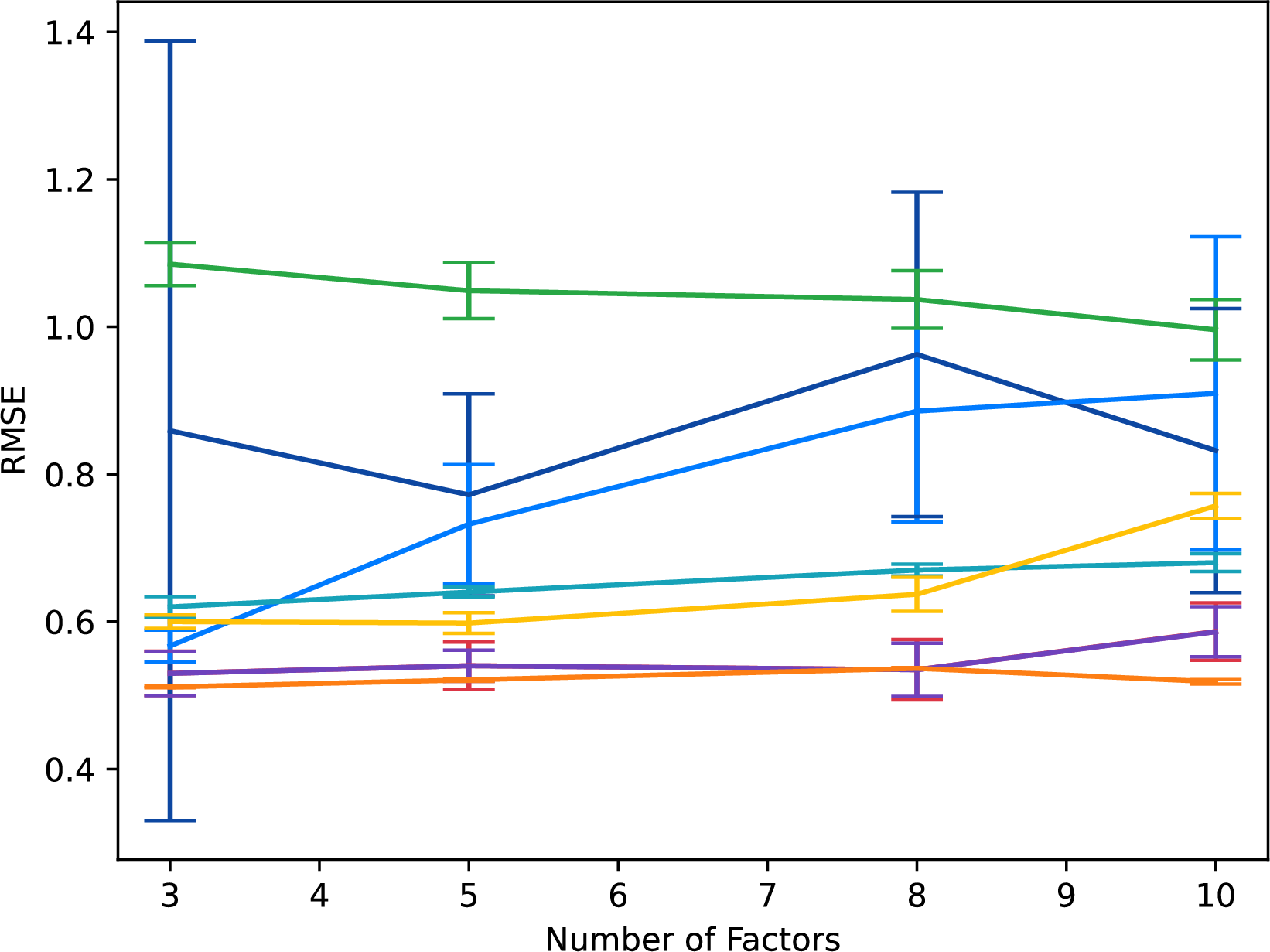

Supplement: S5 Fig — The results are averaged over 5 runs. The performance of several baselines are incomplete, because they are far worse than all the other methods and hence not included. (ZIP) [file pone.0312723.s005.zip › S5 Fig.(b).tif]

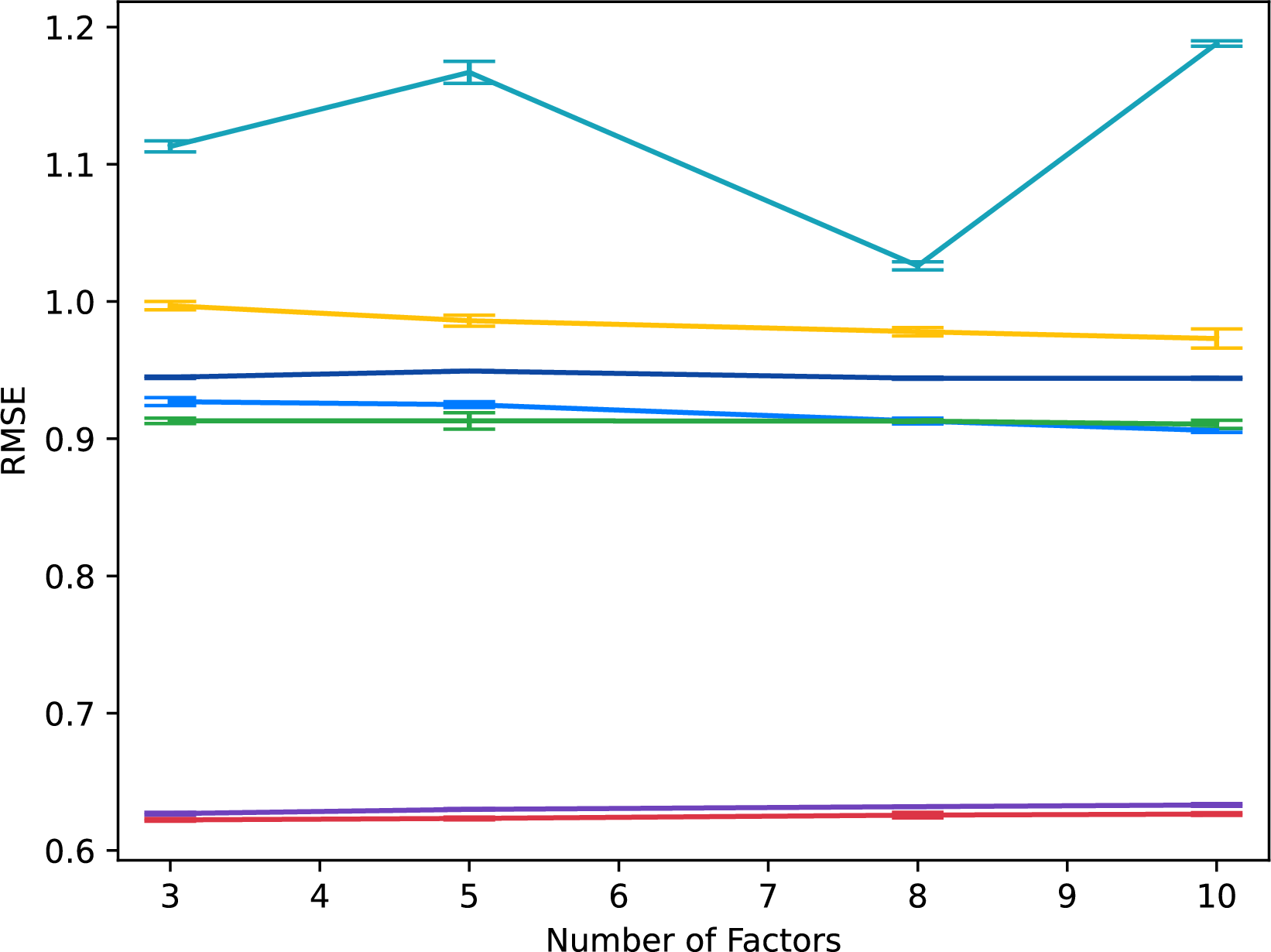

Supplement: S5 Fig — The results are averaged over 5 runs. The performance of several baselines are incomplete, because they are far worse than all the other methods and hence not included. (ZIP) [file pone.0312723.s005.zip › S5 Fig.(c).tif]

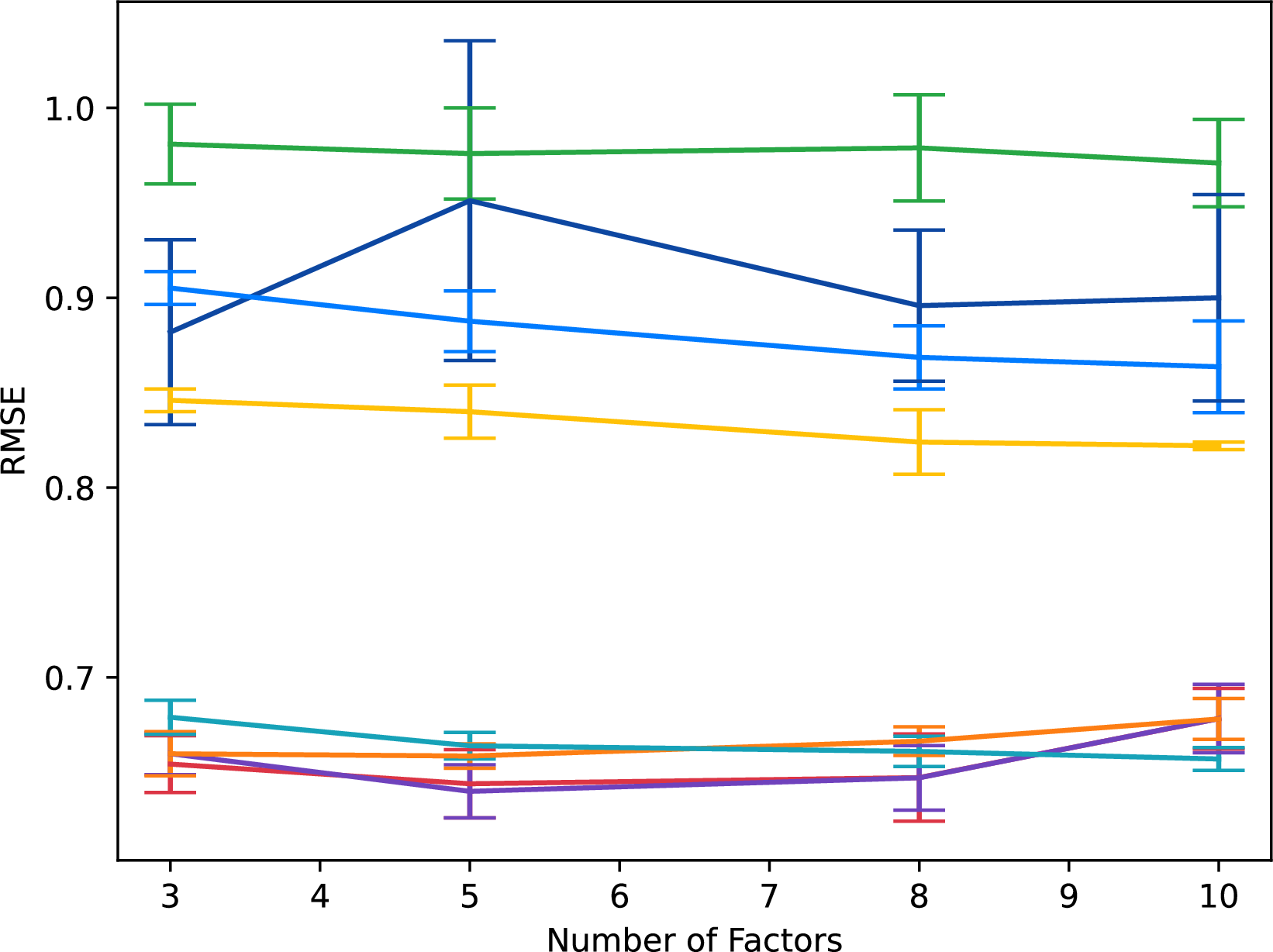

Supplement: S5 Fig — The results are averaged over 5 runs. The performance of several baselines are incomplete, because they are far worse than all the other methods and hence not included. (ZIP) [file pone.0312723.s005.zip › S5 Fig.(d).tif]

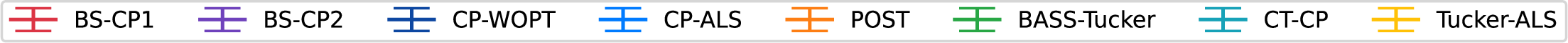

Supplement: S5 Fig — The results are averaged over 5 runs. The performance of several baselines are incomplete, because they are far worse than all the other methods and hence not included. (ZIP) [file pone.0312723.s005.zip › S5 Fig.label.tif]

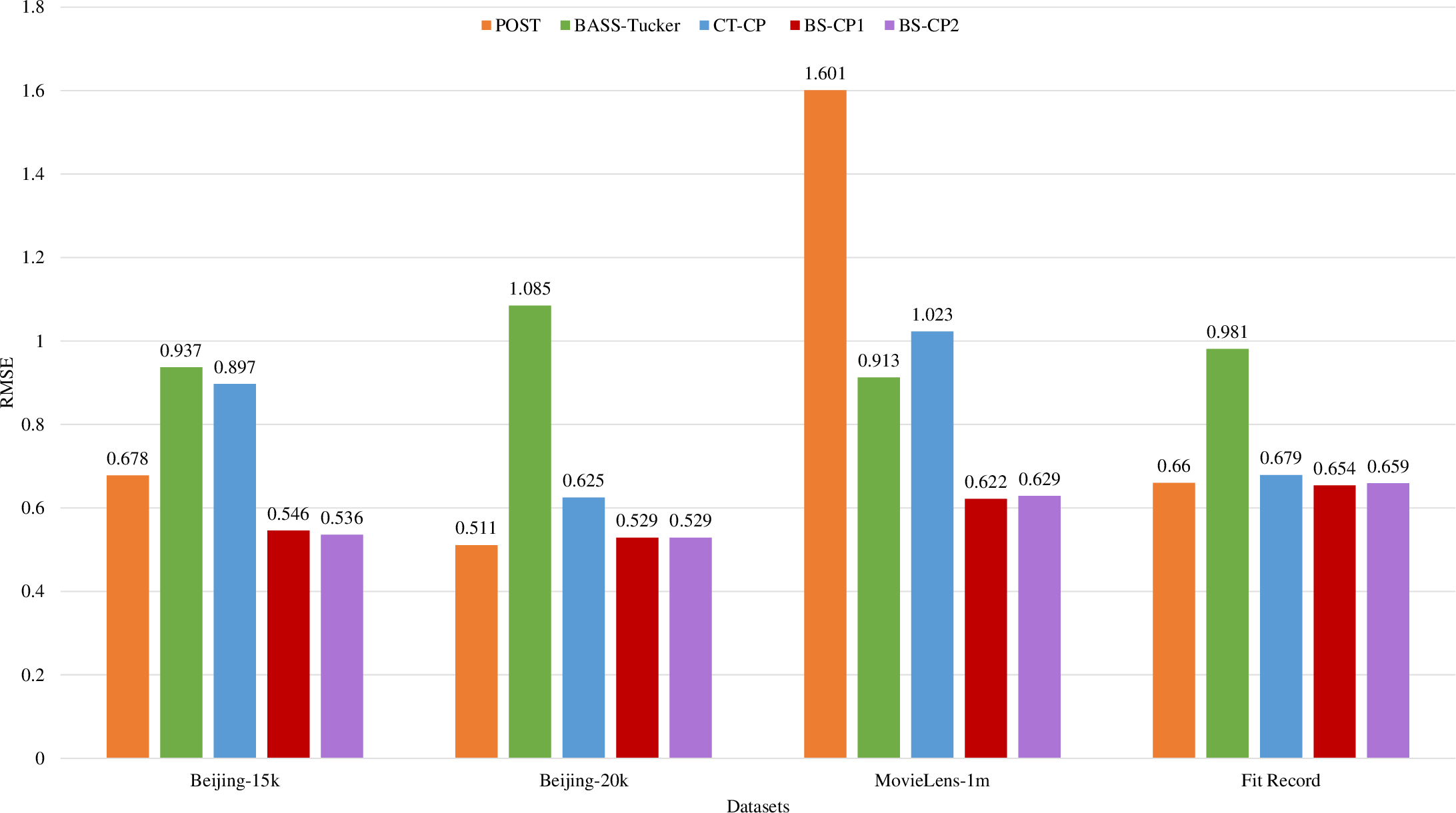

Supplement: S6 Fig — (TIF) [file pone.0312723.s006.tif]

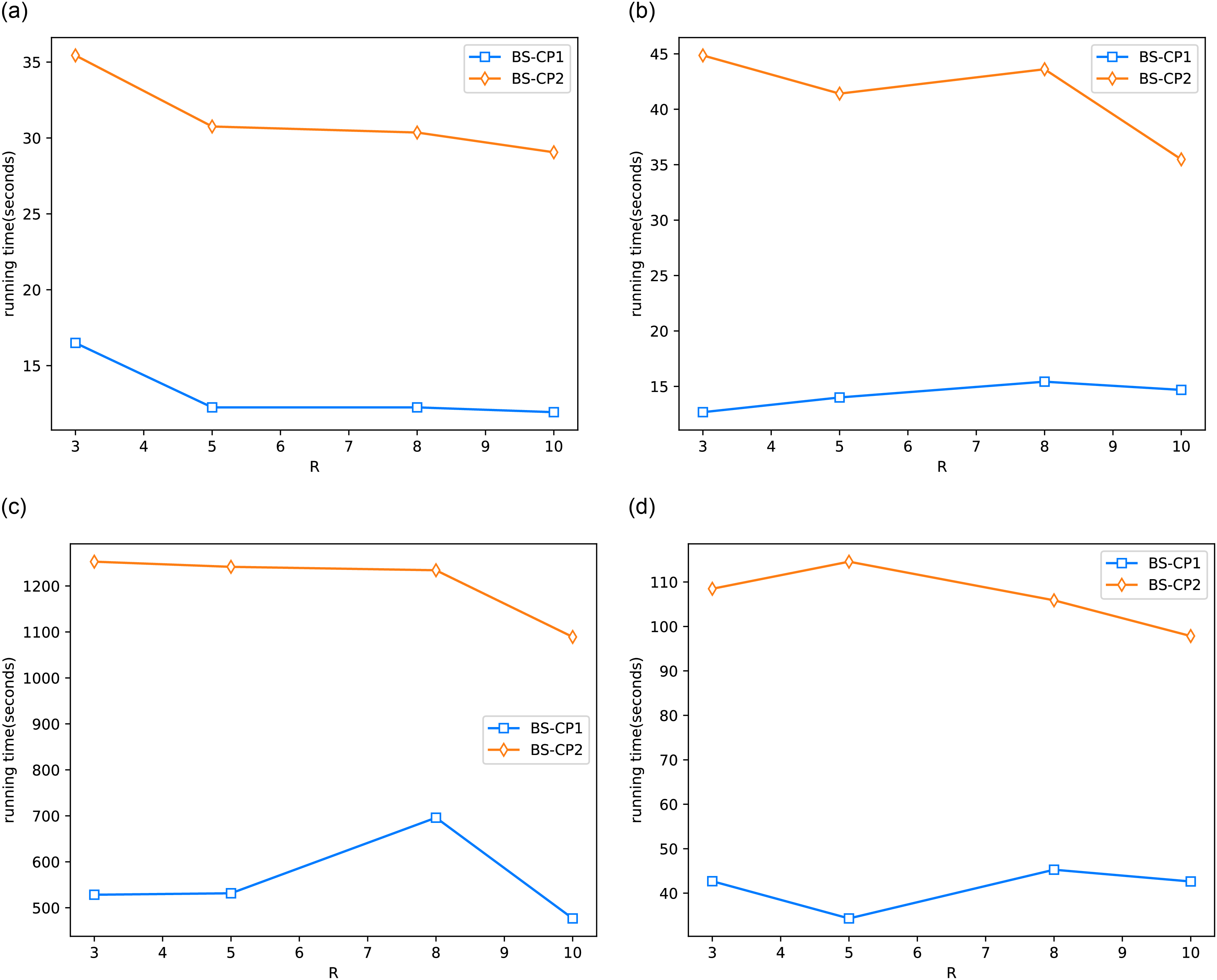

Supplement: S7 Fig — (TIF) [file pone.0312723.s007.tif]

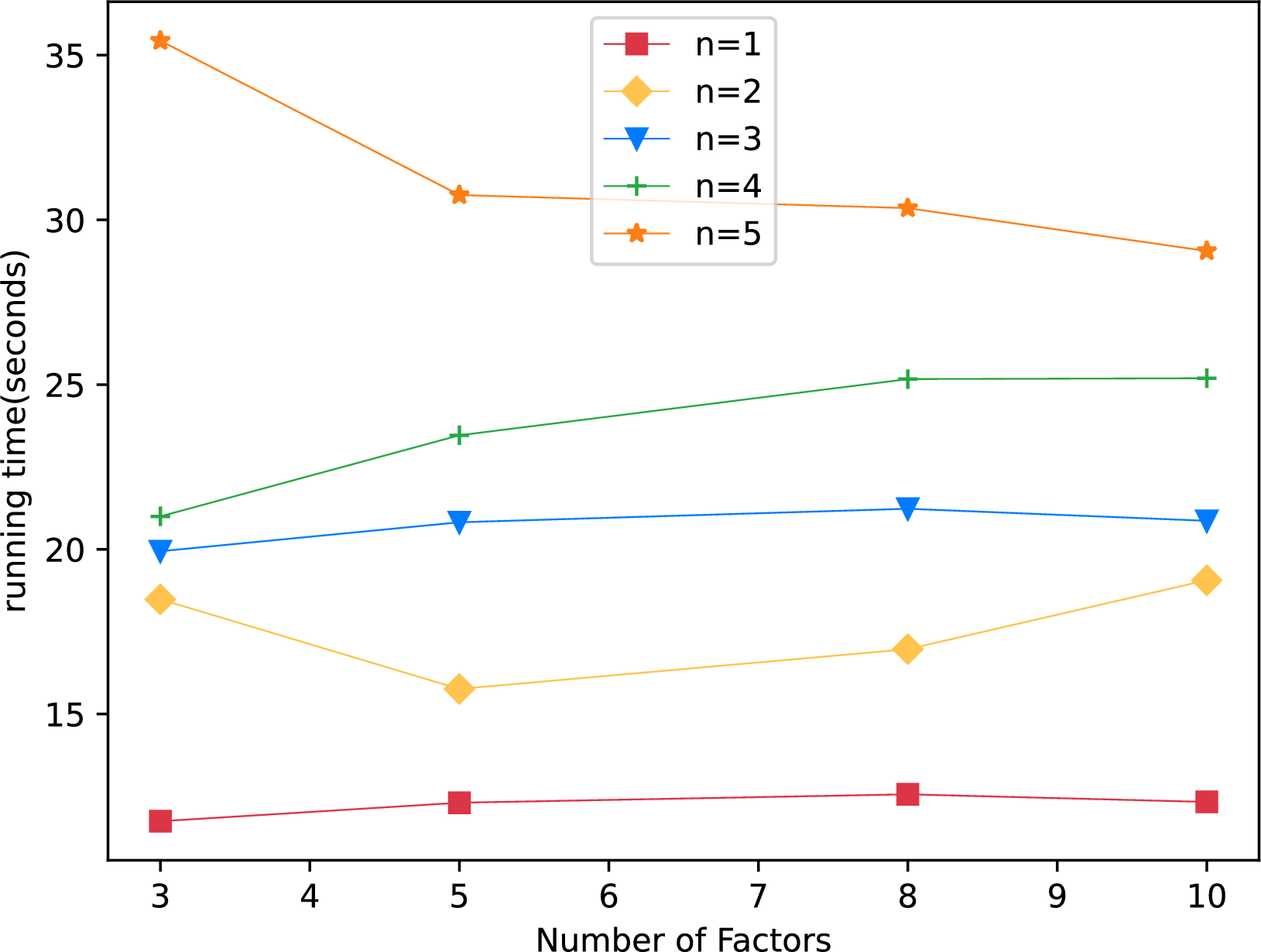

Supplement: S8 Fig — (TIF) [file pone.0312723.s008.tif]

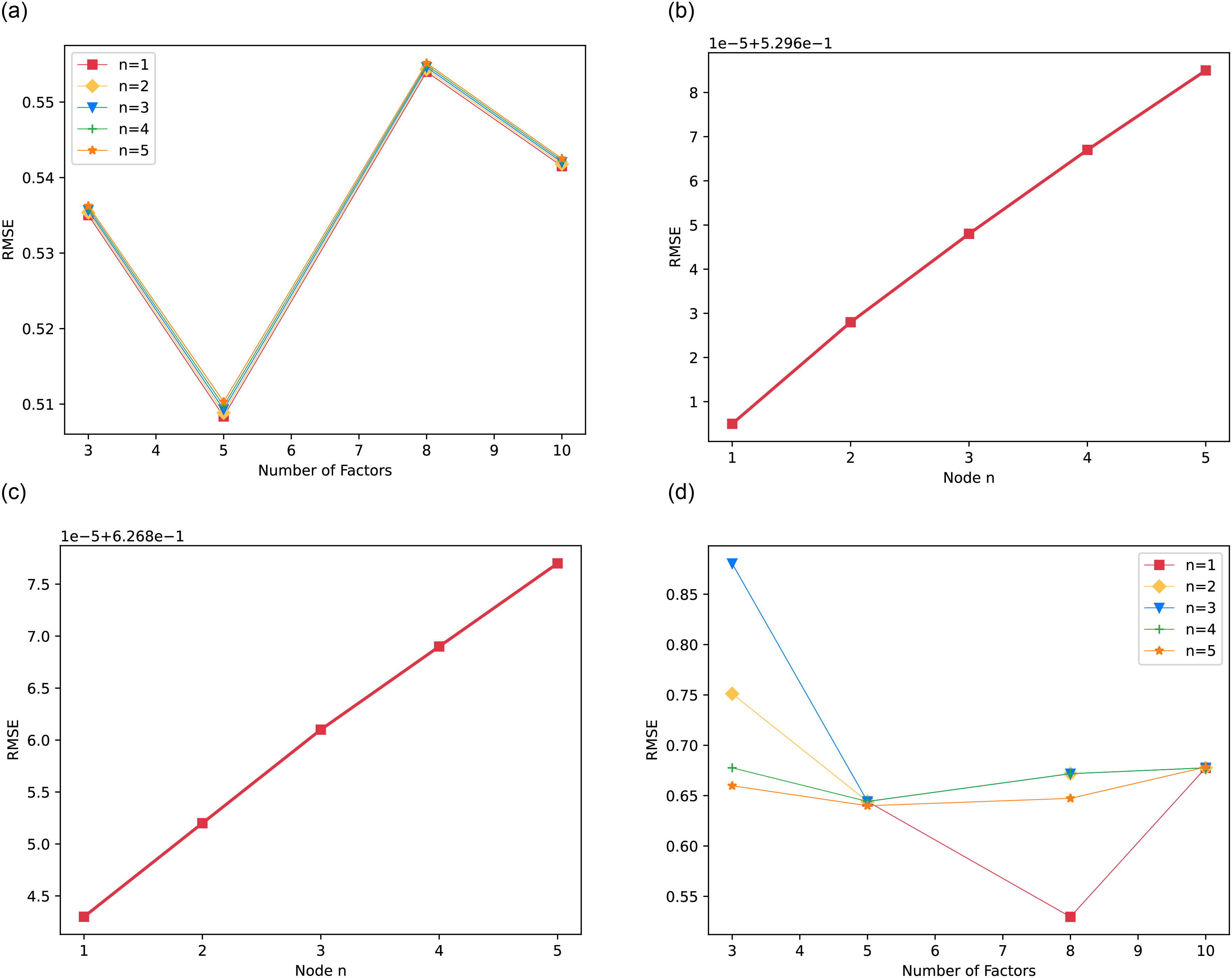

Supplement: S9 Fig — Note that some points have no value because the value is NAN. (b) and (c) are RMSE when R = 3 because the difference between the RMSE values of the two sets of data is too small, we chose an obvious drawing way. (TIF) [file pone.0312723.s009.tif]
